# The PI3K Inhibitor XH30 Enhances Response to Temozolomide in Drug-Resistant Glioblastoma *via* the Noncanonical Hedgehog Signaling Pathway

**DOI:** 10.3389/fphar.2021.749242

**Published:** 2021-11-26

**Authors:** Ming Ji, Zhihui Zhang, Songwen Lin, Chunyang Wang, Jing Jin, Nina Xue, Heng Xu, Xiaoguang Chen

**Affiliations:** ^1^ State Key Laboratory of Bioactive Substances and Functions of Natural Medicines, Institute of Materia Medica, Chinese Academy of Medical Sciences and Peking Union Medical College, Beijing, China; ^2^ Beijing Key Laboratory of Non-Clinical Drug Metabolism and PK/PD Study, Institute of Materia Medica, Chinese Academy of Medical Sciences and Peking Union Medical College, Beijing, China; ^3^ Beijing Key Laboratory of New Drug Mechanisms and Pharmacological Evaluation Study, Institute of Materia Medica, Chinese Academy of Medical Sciences and Peking Union Medical College, Beijing, China

**Keywords:** glioblastoma, TMZ, PI3K, hedgehog, GLI1

## Abstract

Glioblastoma multiforme (GBM) is the most common malignant tumor of the central nervous system. Temozolomide (TMZ)–based adjuvant treatment has improved overall survival, but clinical outcomes remain poor; TMZ resistance is one of the main reasons for this. Here, we report a new phosphatidylinositide 3-kinase inhibitor, XH30; this study aimed to assess the antitumor activity of this compound against TMZ-resistant GBM. XH30 inhibited cell proliferation in TMZ-resistant GBM cells (U251/TMZ and T98G) and induced cell cycle arrest in the G1 phase. In an orthotopic mouse model, XH30 suppressed TMZ-resistant tumor growth. XH30 was also shown to enhance TMZ cytotoxicity both *in vitro* and *in vivo*. Mechanistically, the synergistic effect of XH30 may be attributed to its repression of the key transcription factor GLI1 via the noncanonical hedgehog signaling pathway. XH30 reversed sonic hedgehog–triggered GLI1 activation and decreased GLI1 activation by insulin-like growth factor 1 via the noncanonical hedgehog signaling pathway. These results indicate that XH30 may represent a novel therapeutic option for TMZ-resistant GBM.

## Introduction

Glioblastoma multiforme (GBM) is the most aggressive tumor of the central nervous system in adults (DeAngelis, 2001). Despite recent research focusing on various glioblastoma therapies, the clinical benefit from treatment for people with GBM remains unsatisfactory (Touat et al., 2017; Castresana and Melendez, 2021; Liau, 2021). Mean overall survival is currently estimated to be only 9.7 months after primary treatment.

Temozolomide (TMZ) chemotherapy is still recommended as the standard care for GBM. However, GBM recurs in most people with this tumor type after primary treatment. The development of resistance to TMZ is often the primary limiting factor for treatment success (Bocangel et al., 2002; Lee, 2016; Dymova et al., 2021). Many factors contribute to TMZ resistance, such as overexpression of O6-methylguanine methyltransferase (MGMT), lack of a DNA repair pathway, activation of the hedgehog signaling pathway, the presence of glioma stem cells, and metabolic dysfunction (Happold and Weller, 2015; Perazzoli et al., 2015; Yun et al., 2020; Chien et al., 2021; Zheng et al., 2021). The hedgehog signaling pathway has been a focus of research in this area recently (Melamed et al., 2018; Avery et al., 2021). The glioma-associated oncogene GLI, a zinc finger protein, is a key component in this pathway. Pharmacological inhibition of GLI-1 has been shown to enhance the cytotoxicity of TMZ in GBM and overcome TMZ resistance (Li J. et al., 2016; Ji et al., 2018).

The phosphatidylinositide 3-kinase (PI3K) pathway is frequently overactivated in glioblastoma due to PIK3CA mutations, loss of phosphotase and tensin homolog (*PTEN*) gene function, and amplification of epidermal growth factor receptor (*EGFR*) gene expression (Sami and Karsy, 2013; Langhans et al., 2017; Colardo et al., 2021). PI3K is an attractive therapeutic target for glioblastoma (Li X. et al., 2016; Colardo et al., 2021). In animal models, PI3K inhibitors have been shown to have substantial antitumor activity against GBM (Zhao et al., 2017). The effects of PI3K inhibitors, such as paxalisib, have been tested in patients with newly diagnosed GBM (Wen et al., 2019). Preliminary data have shown encouraging survival outcomes in these patients. In addition, PI3K inhibitors have also been shown to enhance TMZ cytotoxicity in GBM via distinct mechanisms, such as downregulation of ATP-binding cassette subfamily E member 1, inhibition of autophagy, promotion of apoptosis, and inhibition of DNA double-strand break repair (Gil del Alcazar et al., 2014; Radoul et al., 2016; Zhang et al., 2018; Zajac et al., 2021). However, the antitumor activity of PI3K inhibitors in patients with TMZ-resistant GBM currently remains unclear (Hainsworth et al., 2019).

Many studies have reported crosstalk between the PI3K and hedgehog signaling pathways (Ranjan and Srivastava, 2017). The PI3K signaling pathway is a crucial non-canonical activator of GLI1 (Riobo et al., 2006; Zhou et al., 2016; Liang et al., 2017; Ranjan and Srivastava, 2017). Activation of this pathway has been found to enhance GLI1 protein stability because the serine/threonine kinase in this pathway, AKT, can extend the half-life of GLI proteins in the cells by alleviating the inhibitory effect of protein kinase A, thus facilitating nuclear translocation (Singh et al., 2017). Meanwhile, PI3K signaling activates GLI1 via its downstream effector, ribosomal S6 kinase (p70S6K) (Wang et al., 2012). Activated p70S6K promotes GLI1 disassociation from suppressor of fused homolog (SUFU) by phosphorylating GLI1 at Ser84 and enhancing GLI1 transcriptional activity. In addition, p70S6K2 has been shown to inhibit glycogen synthase kinase (GSK3) by phosphorylating GLI1 at Ser9, leading to a decrease in GSK3β-mediated GLI1 degradation (Mizuarai et al., 2009).

XH30 is a PI3K inhibitor that can cross the blood–brain barrier (Lin et al., 2018). XH30 has previously been demonstrated to have robust antitumor activity in GBM and brain metastases of lung cancer *in vivo* (Lin et al., 2018; Ji et al., 2021). In this study, we aimed to assess the capacity of XH30 to inhibit the growth of GBM cells with natural or TMZ-induced drug resistance both *in vitro* and *in vivo*, in an orthotopic mouse model. We also explored the underlying mechanisms of the antitumor effects of XH30.

## Materials and Methods

### Cell Lines

Two cell lines were used in this study. The T98G human glioma tumor cell line was purchased from ATCC (Manassas, VA, United States); this is a naturally TMZ-resistant cell line with elevated levels of MGMT. The U251/TMZ cell line was a gift from Dr. Yuhui Zou of General Hospital of Guangzhou Military Command of People’s Liberation Army, as previously reported (Ji et al., 2018); this line has acquired TMZ resistance, with overactivated hedgehog signaling. Both cell lines were cultured in Dulbecco’s modified eagle medium (Gibco, TX, United States) with 10% (v/v) fetal bovine serum (Gibco), penicillin (100 units/ml), and streptomycin (100 units/ml) in a humidified atmosphere of 5% CO_2_ at 37°C.

### Antibodies and Reagents

XH30 was synthesized in house as described previously (Lin et al., 2018). PI3K inhibitor PF-04691502 was purchased from Selleck Chemicals (Houston, TX, United States). TMZ was purchased from J&K Scientific (Beijing, China). All antibodies [AKT, phosphor-AKT (S473), phosphor-AKT (T308), mammalian target of rapamycin (mTOR), phosphor-mTOR (S2448), GSK3β, phosphor-GSK3β (S9), proline-rich AKT substrate of 40 kDa (PRAS40), phosphor-PRAS40 (T246), p70S6K, phosphor-p70S6K (T389), S6 ribosomal protein (S6RP), phosphor-S6RP (S240/244), smoothened (SMO), cyclin D1, and anti-cyclin–dependent kinase (CDK2)] were purchased from Cell Signaling Technology (Danvers, MA, United States). Anti-GLI1 and anti–β-actin antibodies were purchased from Abcam (Cambridge, United Kingdom) and Santa Cruz Biotechnology (Dallas, TX, United States), respectively.

### Cell Viability Assay

Cell viability was assessed using the Cell Counting Kit-8 (CCK-8; Solarbio, Beijing, China). Briefly, 2,000 cells per well were seeded into a 96-well plate. After incubation overnight, the cells were treated with different concentrations (0.0512, 0.256, 1.28, 6.4, 32, 160, 800, 4,000, and 20,000 nM) of XH30 or PF-04691502 with three replicates for 72 h. Then, CCK-8 solution was added and incubated for 3 h, after which, absorbance values were measured at 450 nM using a microplate reader (BioTek Instruments, Inc., United States). The half-maximal inhibitory concentration (IC_50_) was calculated using GraphPad Prism v8.0.1 (La Jolla, CA, United States).

### Colony Formation Assay

U251/TMZ and T98G cells were seeded at a density of 200 cells per well into six-well plates. After 24 h, the cells were treated with indicated concentrations (4, 20, 100, and 500 nM) of XH30 with three replicates. The culture medium with test compound was replaced every 3 days. After cell colonies formed, cells were washed with phosphate-buffered saline (PBS) and fixed with 4% paraformaldehyde for 15 min, then stained with crystal violet for 30 min, and washed with PBS. Finally, colonies were recorded with a photograph and measured by a microplate reader (BioTek).

### Cell Cycle Analysis

Flow cytometry assays were used to analyze the cell cycle distribution as previously reported (Ji et al., 2018). In brief, U251/TMZ and T98G cells were dispensed into six-well plates at a density of 50,000 cells per well. After growing overnight in a humidified atmosphere of 5% CO_2_ at 37°C, cells were treated with indicated concentrations (4, 20, 100, and 500 nM) of XH30 for 24 h. Then, cells were harvested and fixed ice cold 70% ethanol overnight at –20°C, washed with PBS, and stained with propidium iodide (PI) solution containing PI (20 mg/ml) and RNase A (20 mg/ml) in PBS for 30 min. DNA contents were measured using the BD fluorescence-activated cell sorting verse flow cytometer (BD Biosciences, NJ, United States), and the cell cycle distribution was analyzed.

### Immunoblotting Analysis

Cells or mice tumor tissues were collected and lysed in RIPA lysate buffer supplemented with 1% protease inhibitor cocktail and 1% phosphatase inhibitor cocktail (Solarbio, Beijing, China). Lysates were then centrifuged at 12,000 g for 30 min. Proteins were quantified using a bicinchoninic acid assay kit (Solarbio, Beijing, China). Resultant samples containing equal amounts of proteins were subjected to sodium dodecylsulfate–polyacrylamide gel electrophoresis and transferred to a polyvinylidene fluoride membrane (Millipore, Darmstadt, Germany). The membrane was blocked with TBST buffer containing 5% non-fat milk for 30 min and incubated with appropriate primary antibodies (1:1,000 dilution) in TBST at 4°C overnight. After washing with TBST, the membrane was incubated with horseradish peroxidase–conjugated secondary antibodies (1:2,000 dilution; Cell Signaling Technologies, Boston, MA) for 1 h at room temperature. Bound proteins were visualized using enhanced chemiluminescence and detected using ImageQuant LAS 4000 software.

### Quantitative Real-Time Polymerase Chain Reaction Analysis

Total RNA from XH30-treated U251/TMZ or T98G cells was isolated using TRIzol reagent (Bioteke Corporation, China) according to the recommended procedures of the manufacturer. First-strand cDNA was synthesized from 1 μg of total RNA using the ReverTra Ace^®^ qPCR RT Master Mix with gDNA Remover (Toyobo, Japan). Real-time polymerase chain reaction (PCR) was performed using the Analytikjena qTOWER detection system and the SYBR^®^ Green RT-PCR master mix (Toyobo). Target sequences were amplified at 95°C for 1 min, followed by 40 cycles at 95°C for 15 s, 60°C for 15 s, and 72°C for 45 s. Fold changes in *GLI1*, paired box protein 6 (*PAX6*), and O6-methylguanine-DNA-MGMT (*MGMT*) gene expression were calculated according to the 2−ΔΔCt method. The primers sequences used to amplify specific regions of the indicated genes were as follows: *GLI1* forward, ATG​TTC​AAC​TCG​ATG​ACC​CCA​C; *GLI1* reverse, CAA​CTT​GAC​TTC​TGT​CCC​CAC​A; *MGMT* forward, ATGGAT GTTTGAGCGACACA; *MGMT* reverse, ATA​GAG​CAA​GGG​CAG​CGT​TA; *PAX6* forward, AAC​GAT​AAC​ATA​CCA​AGC​GTG​T; *PAX6* reverse, GGT​CTG​CCC​GTT​CAA​CAT​C.

### Orthotopic Mouse Tumor Model and Subcutaneous Mouse Tumor Model

Eight- to 10-week-old female Balb/c athymic nude mice (SPF Biotechnology, Beijing, China) were housed in standard facilities. Human U251/TMZ cells in PBS were injected intracranially, 2.0 mm below the skull surface, according to a previously published protocol (Ji et al., 2018). Three days after surgery, mice were randomized to receive one of the four following treatments: a drug vehicle (delivered orally once per day for 9 days). TMZ (50 mg/kg, delivered orally once per day for 5 days), or XH30 (5 mg/kg, delivered orally once per day for 9 days). TMZ and XH30 were dissolved in 0.5% carboxymethylcellulose solution. Tumor volumes were monitored using an animal magnetic resonance imaging (MRI) scanner (PharmaScan 70/16 US, Bruker, Germany). The parameters for the MRI scans were as follows: a T2_TurboRARE, with TR/TE = 5,000/40, 6 averages, 20 × 20 field of view, and 0.5-mm slice thickness. The tumor volume on the basis of MRI was calculated as V = L × W × T, where L is the maximum length of tumor, W is the maximum width perpendicular to L, and T is the thickness of the tumor slice (set at 0.5 mm).

For the subcutaneous mice tumor model, female Balb/c athymic nude mice (eight to 10 weeks of age) were subcutaneously implanted with 1 × 10^7^ U251/TMZ cells in 0.2 ml matrigel solution in the right flank. After 2 weeks, tumor issue was harvested sterilely, and tumor cells were extracted from the tissue homogenate. Then, the mice were implanted with 2 × 10^6^ tumor cells each in the right flank. Seven days later, when the average tumor volumes reached to 100–300 mm^3^, the mice were randomized into four groups, in which either alone treatment or combination was administered, respectively, using the same dose regime as in the orthotopic model. Tumor volume and body weight were monitored twice a week. Tumor volume was calculated as V = 1/2 × L × W^2^, where L is the maximum length of tumor and W is the maximum width of tumor. The mice were euthanized at day 14, and tumor tissues were collected for immunoblotting.

All procedures were approved by the Ethics Committee for Animal Experiments of the Institute of Materia Medica, Chinese Academy of Medical Sciences and Peking Union Medical College and conducted under the Guidelines for Animal Experiments of Peking Union Medical College.

### Statistical Analysis

Most statistical analyses were performed utilizing GraphPad Prism v8.0.1 (La Jolla, CA, United States), and significance levels were evaluated using analysis of variance (ANOVA) or T-tests, as appropriate. In our experiments, we distinguish between three of significance (***p < 0.001, **p < 0.01, and *p < 0.05, respectively).

On the basis of the cell viability assay, the combination index (CI) was calculated using the Chou–Talalay method, with CI = 1, CI < 1 and CI > 1 denoting an additive effect, synergism, and antagonism, respectively. CI = (D)1/(Dx)1 + (D)2/(Dx)2, where (Dx)1 and (Dx)2 represented concentrations of each drug alone to exert x% effect, while (D)1 and (D)2 were concentrations of drugs in combination to elicit the same effect.

## Results

### The PI3K Inhibitor XH30 Inhibited TMZ-Resistant GBM Cell Growth *in vitro*

**FIGURE 1:**
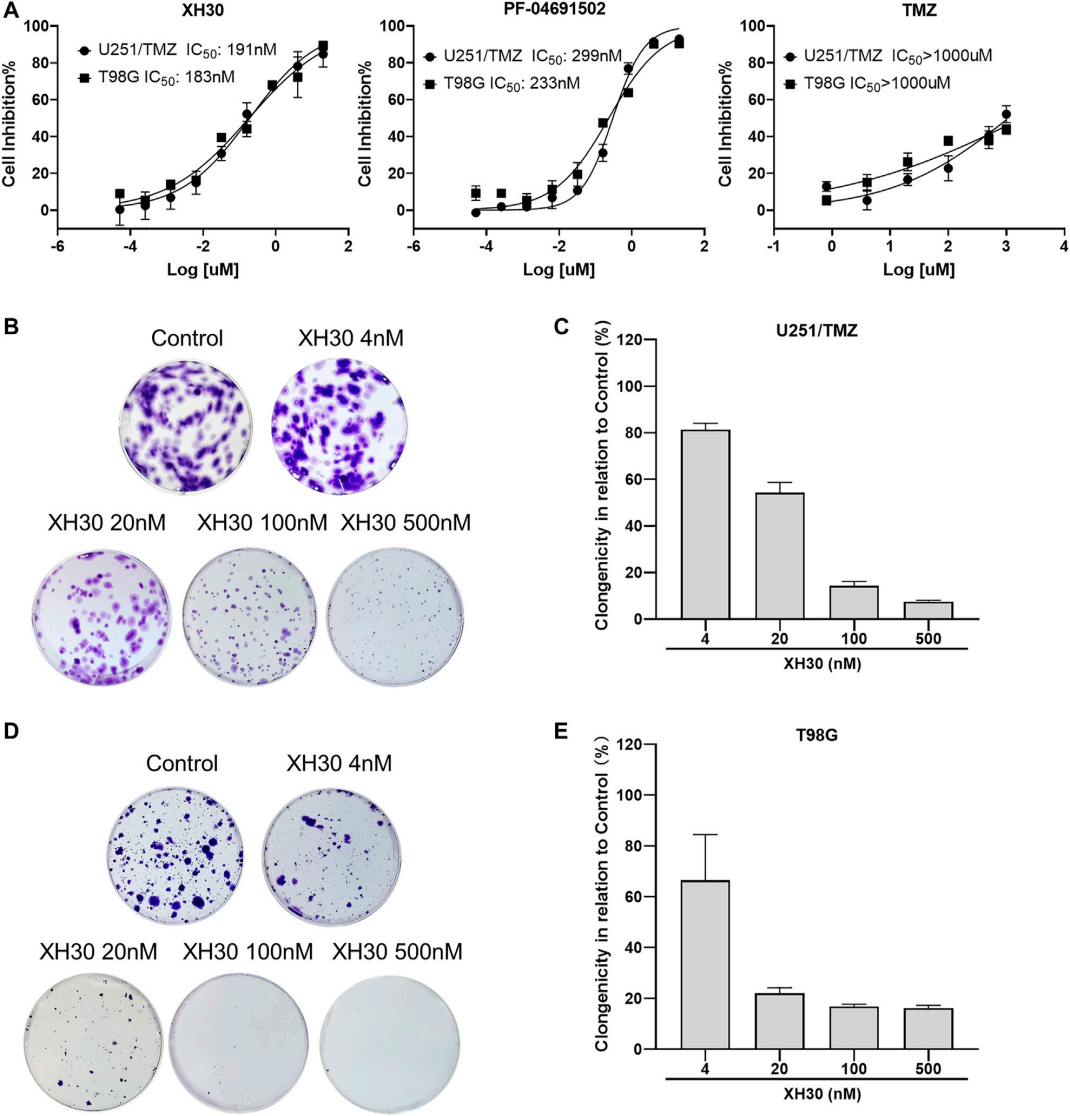
The PI3K inhibitor XH30 inhibited cell proliferation in temozolomide (TMZ)–resistant glioblastoma multiforme (GBM) cells. **(A)** The IC_50_ values of XH30, PF-04691502 (control), and TMZ in TMZ-resistant GBM cells over 72 h. Data are presented as means ± SD, *n* = 3. **(B,D)** Colony formation assay of XH30 in TMZ-resistant U251/TMZ and T98G cells, respectively. Representative images are shown for each group. **(C,E)** The inhibition ratio of colony formation assay of XH30 in U251/TMZ and T98G cells, respectively. Data are presented as means ± SD, *n* = 3.

Here, we assessed the anti-tumor activity of XH30 in two TMZ-resistant GBM cell lines: one with acquired TMZ resistance and overactivated hedgehog signaling (U251/TMZ) and one with natural TMZ resistance and elevated MGMT levels (T98G). As shown in Figure 1A, TMZ did not exhibit cytotoxicity at the highest concentration of 1,000 μM. XH30 suppressed cell proliferation of both U251/TMZ and T98G cells, with 72 h IC_50_ values of 191 and 183 nM, respectively. The cytotoxic effect was stronger at 72 h than at 24 or 48 h (Supplementary Figure S1). The positive control, PF-04691502, also inhibited the proliferation of both cell types. In a colony formation assay, the formation of cell colonies dose-dependently decreased after exposure to XH30 in both U251/TMZ and T98G cells (Figures 1B–E). These results indicated that XH30 exhibited strong inhibitory effects on the proliferation of TMZ-resistant GBM cells.

### XH30 Reduced Downstream Molecules in the PI3K Signaling Pathway in TMZ-Resistant GBM Cells

**FIGURE 2:**
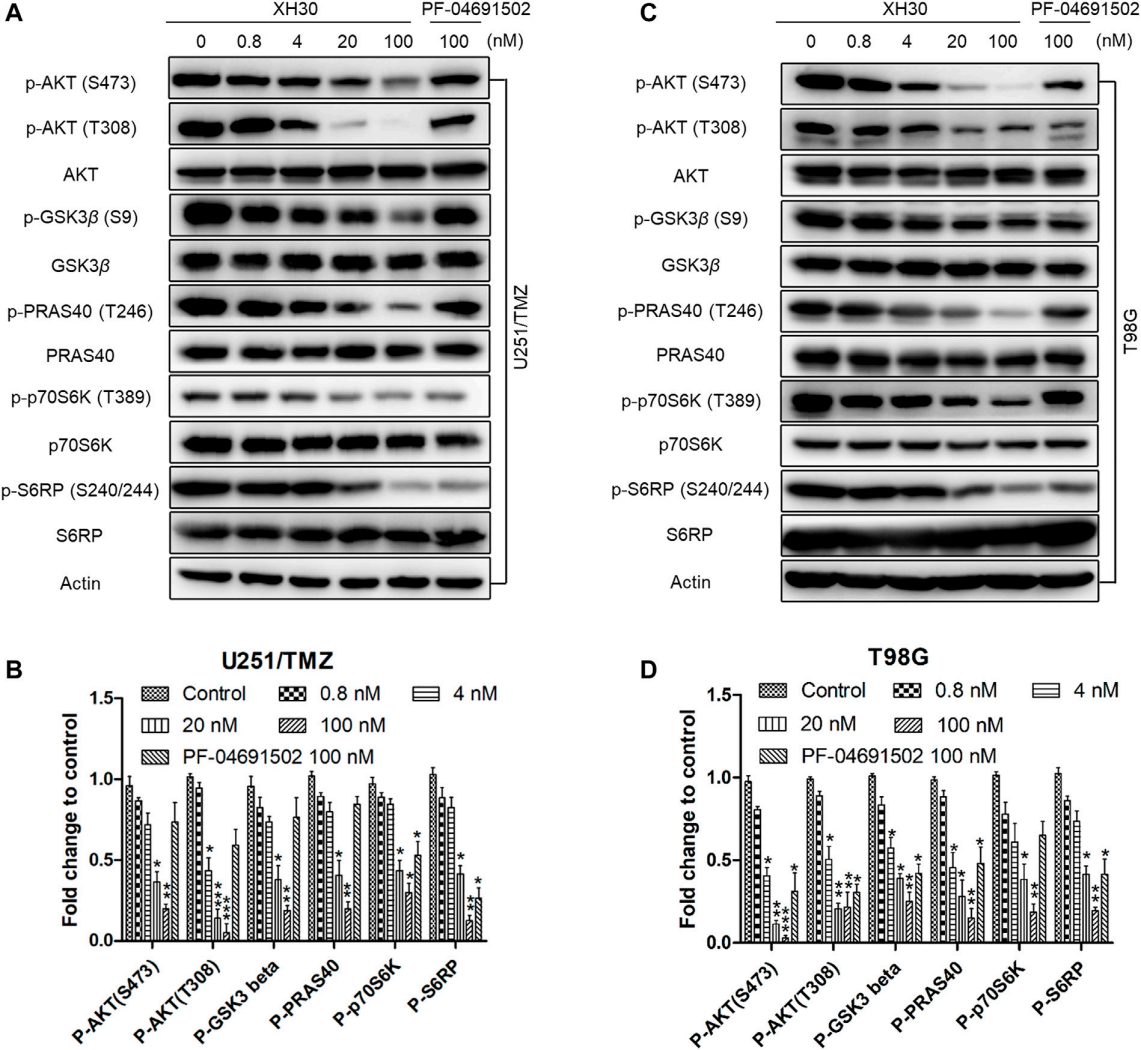
The PI3K inhibitor XH30 suppressed the PI3K signaling pathway in TMZ-resistant GBM cells. **(A)** XH30 dose-dependently inhibited PI3K pathway signaling in TMZ-resistant U251/TMZ cells. The cells were incubated with XH30 at indicated concentrations (0.8, 4, 20, and 100 nmol/L) or PF-04691502 (100 nmol/L) as a control for 3 h. The experiment was repeated three times. **(B)** Relative expression levels of the major proteins in **(A)**. Data are presented as means ± SD, *n* = 3. *t*-test, ^*^ *p* < 0.05, ^**^ *p* < 0.01, and ^***^ *p* < 0.001 compared with control. **(C)** XH30 dose-dependently inhibited PI3K pathway signaling in TMZ-resistant T98G cells. The cells were incubated with XH30 at indicated concentrations (0.8, 4, 20, and 100 nmol/L) or PF-04691502 (100 nmol/L) as a control for 3 h. The experiment was repeated three times. **(D)** Relative expression levels of major proteins in **(C)**. Data are presented as means ± SD, *n* = 3. *t*-test, ^*^ *p* < 0.05, ^**^ *p* < 0.01, and ^***^ *p* < 0.001, compared with control.

We assessed the inhibitory activity of XH30 on the PI3K signaling pathway in both U251/TMZ and T98G cells. XH30 dose-dependently blocked downstream molecules in the PI3K pathway including p-AKT, p-GSK3β, p-PRAS40, p-p70S6K, and p-S6RP (Figures 2A–D). At a concentration of 100 nM, XH30 strongly suppressed the phosphorylation of these signaling molecules. This inhibitory activity was more potent than the inhibitory effect of the positive control, PF-04691502, at the same concentrations.

### XH30 Induced Cell Cycle Arrest in TMZ-Resistant GBM Cells

**FIGURE 3:**
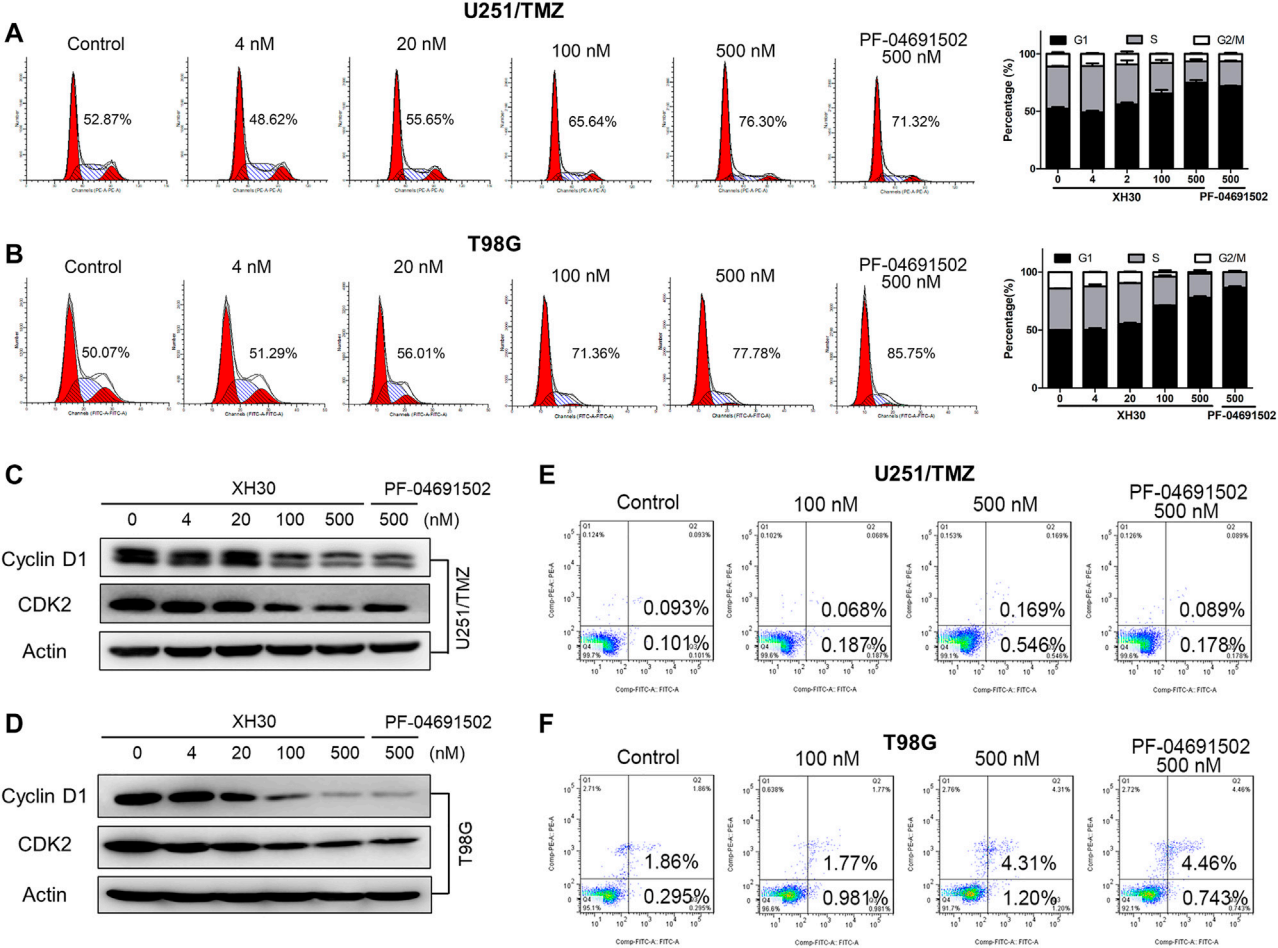
XH30 induced G1 cell-cycle arrest in TMZ-resistant GBM cells. **(A, B)** XH30 induced cell cycle arrest in TMZ-resistant U251/TMZ and T98G cells in the G1 phase. Exposure to various concentrations of XH30 at indicated concentrations (4, 20, 100, and 500 nM) or the control, PF-04691502 (500 nM), for 24 h. The cells were stained with propidium iodide (PI) for flow cytometry analysis, *n* = 3. **(C, D)** XH30 downregulated markers of the G1 phase of the cell cycle. The protein levels of cyclin D1 and CDK2 were detected *via* immunoblotting in both U251/TMZ and T98G cells exposed to XH30 for 24 h. **(E, F)** The effect of XH30 on apoptosis in both U251/TMZ and T98G cells exposed to XH30 for 48 h.

Next, we investigated whether XH30 could induce cell cycle arrest or apoptosis in both TMZ-resistant GBM cell lines. In U251/TMZ cells, both PF-04691502 and XH30 induced cell cycle arrest in the G0/G1 phases. The percentage of G1 phase increased from 48.62% to 76.30% after a concentration titration of XH30 (Figure 3A). In T98MG cells, cell cycle arrest was observed during the G1 phase after exposure to XH30. The percentage of G1 phase at a XH30 concentration of 500 nM was increased compared to the control group (77.78% vs. 50.07%) (Figure 3B). Moreover, XH30 downregulated the expression of cyclin D1 and CDK2, which are markers of the G1 phase (Figures 3C,D). However, apoptosis was not observed in either U251/TMZ or T98G cells after exposure to XH30 for 48 h. These data demonstrated that XH30 induces cell cycle arrest in TMZ-resistant GBM cells (Figures 3E,F).

### XH30 Exhibited Antitumor Activity in TMZ-Resistant GBM *in vivo*

**FIGURE 4:**
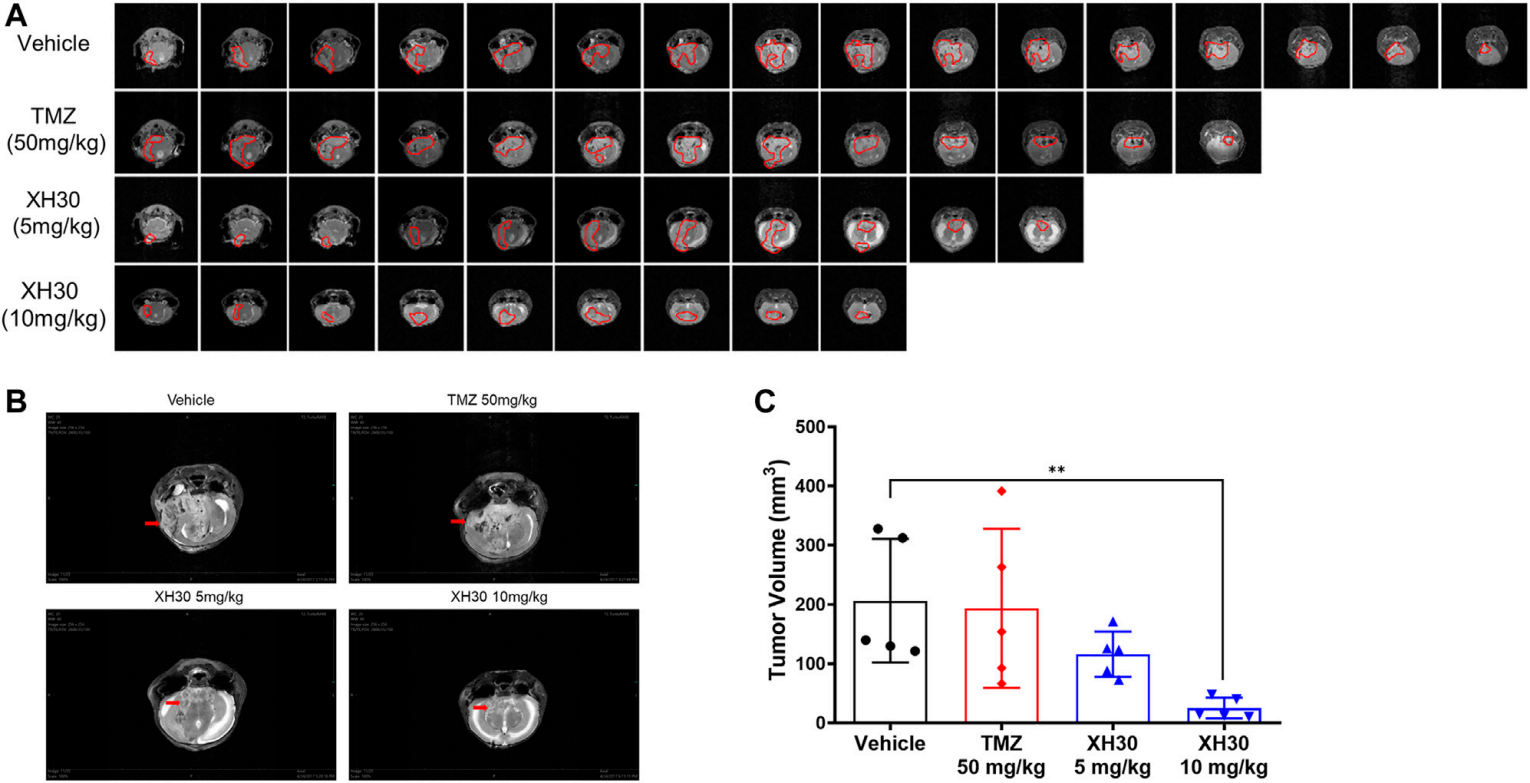
XH30 repressed TMZ-resistant GBM growth in a mouse orthotopic xenograft model. **(A)** magnetic resonance imaging (MRI) T2-weighted image of intracranial tumors from the various groups from the U251/TMZ orthotopic model at day 9. Images in each lane show all tumor slices from one representative mouse in each group. The red curves indicate the tumors. **(B)** Representative MRI images from the U251/TMZ orthotopic model at day 9. The red arrows indicate the tumor. **(C)** Tumor volumes in the U251/TMZ orthotopic model at day 9. Data are presented as means ± SD, *n* = 5. ANOVA, ^**^ *p* < 0.01 compared with the vehicle control group.

To further explore the antitumor activity of XH30 in TMZ-resistant GBM, we used a U251/TMZ orthotopic mouse model. Mice were given TMZ orally at doses of 50 mg/kg/day for 5 days or XH30 at either 5 mg/kg or 10 mg/kg daily for 9 days. In our MRI analysis, the images showed that XH30 suppressed tumor growth in brain (Figures 4A,B). At a dose of 10 mg/kg/day, tumor volume was significantly lower compared to the control group that received the drug vehicle (*p* < 0.01; Figure 4C; Supplementary Table S1). In this model, TMZ did not reduce tumor volume at a dose of 50 mg/kg/day with 6.2% tumor growth inhibition (TGI), whereas the TGI of TMZ at the same dose was 98.4% in a U251 (TMZ sensitive) orthotopic mice model (Supplementary Figures S3A, B). During the experiments, the body weight of mice in the XH30 group did not significantly decrease compared to that of the control group (Supplementary Figure S2A). Together, these data indicate that XH30 suppressed TMZ-resistant GBM growth *in vivo*.

### XH30 Enhanced TMZ Cytotoxicity in TMZ-Resistant GBM

**FIGURE 5:**
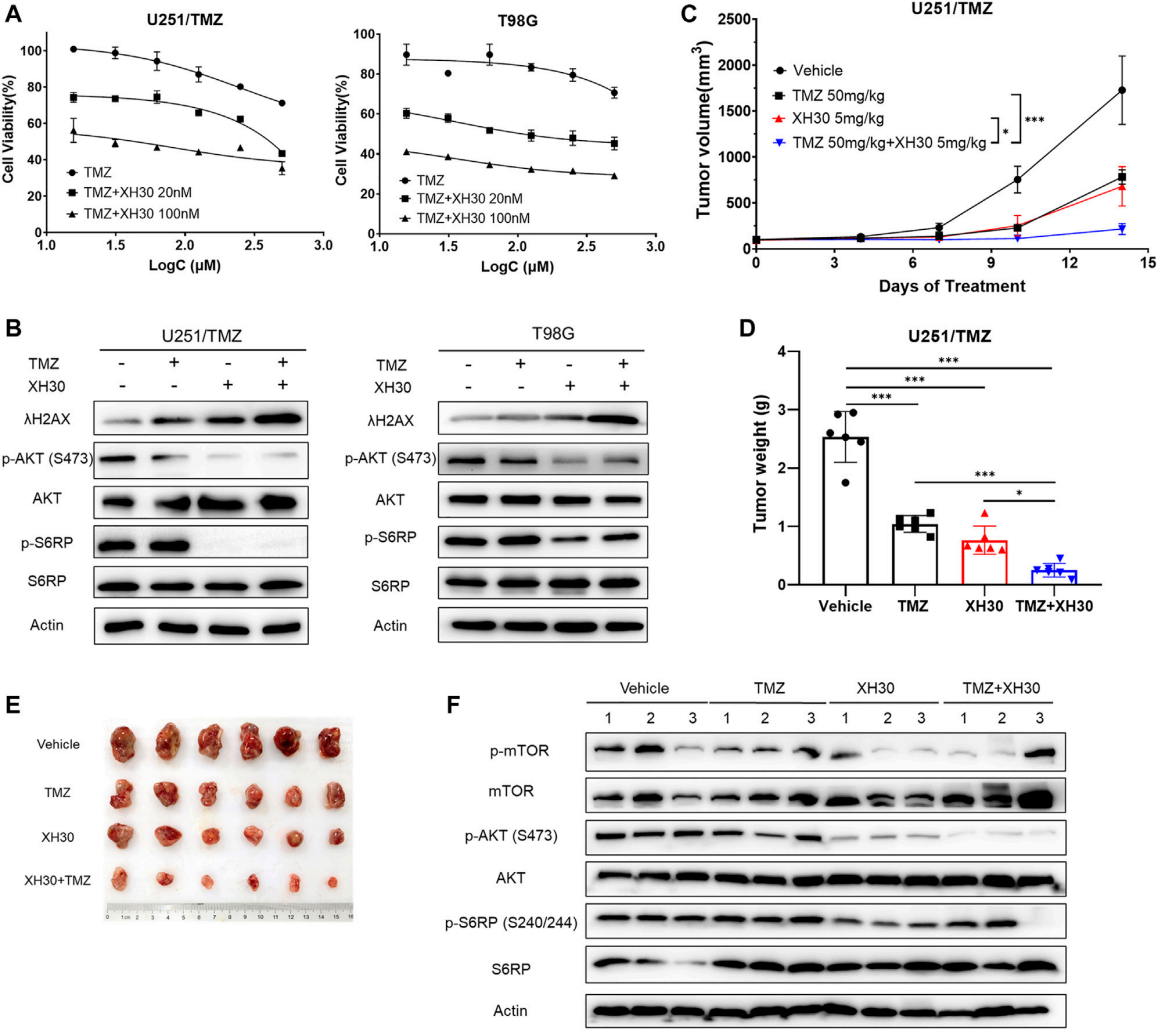
XH30 enhanced TMZ response in TMZ-resistant GBM. **(A)** The curve for combined XH30 and TMZ treatment in GBM cells for 72 h. Data are presented as means ± SD, *n* = 3. **(B)** Combination of XH30 with TMZ increased the level of λH2AX. Cells were incubated with XH30 (100 nM) or TMZ (250 μM) for 12 h. **(C)** Antitumor activity of XH30 in an U251/TMZ subcutaneous xenograft model. *t*-test, ^*^ *p* < 0.05 and ^***^ *p* < 0.001, compared with the TMZ or XH30 group. Data are presented as means ± SD, *n* = 6. **(D)** Tumor weight in an U251/TMZ xenograft model. *t*-test, ^*^ *p* < 0.05 and ^***^ *p* < 0.001. **(E)** Tumor issues in a U251/TMZ xenograft model. **(F)** Levels of proteins in the PI3K signaling pathway in tumor tissue from the U251/TMZ xenograft mice model.

Because XH30 showed excellent antitumor activity against TMZ-resistant GBM and TMZ resistance is a current barrier to effective treatment in GBM, we investigated whether XH30 could sensitize GBM to TMZ. To do this, XH30 and TMZ were administered together in GBM cells. XH30 at concentrations of 20 and 100 nM enhanced TMZ cytotoxicity in both U251/TMZ and T98G cells (Figure 5A). Calculation of the CI gave values of <1 for combined XH30 and TMZ in both cell types, indicating a synergic effect of these drugs (Supplementary Table S2). Immunoblotting results showed that the combination of XH30 and TMZ increased the level of the DNA damage marker λH2AX to a greater extent compared with TMZ or XH30 alone, indicating that XH30 increases TMZ cytotoxicity (Figure 5B).

We then evaluated whether combining XH30 and TMZ had synergistic antitumor effects *in vivo*. We employed a subcutaneous mouse model implanted with U251/TMZ cells. The groups that received XH30 or a combination of XH30 and TMZ both had significantly delayed tumor growth (Figure 5C). Treatment with either TMZ at 50 mg/kg/day or XH30 at 5 mg/kg/day suppressed tumor growth, with TGI values of 58.9% and 69.9%, respectively (Figures 5D,E). Combined TMZ and XH30 significantly suppressed tumor growth compared with the groups that received either TMZ or XH30 alone, with a TGI of 90.1%. Although TMZ exhibited antitumor activity, the effect was weaker than in the parent cell line U251 (TMZ sensitive) in the subcutaneous mouse model (58.9% vs. 94.9% TGI, Figure S3C and D). The body weight change with combined TMZ and XH30 treatment was within acceptable limits (Supplementary Figure S4). The immunoblotting data also showed that the phosphorylation proteins downstream to PI3K including mTOR, AKT, and S6RP decreased in tumor tissues with XH30 alone and in combination with TMZ (Figure 5F).

### XH30 Repressed GLI1 *via* the Noncanonical Hedgehog Signaling Pathway to Increase TMZ Cytotoxicity

**FIGURE 6:**
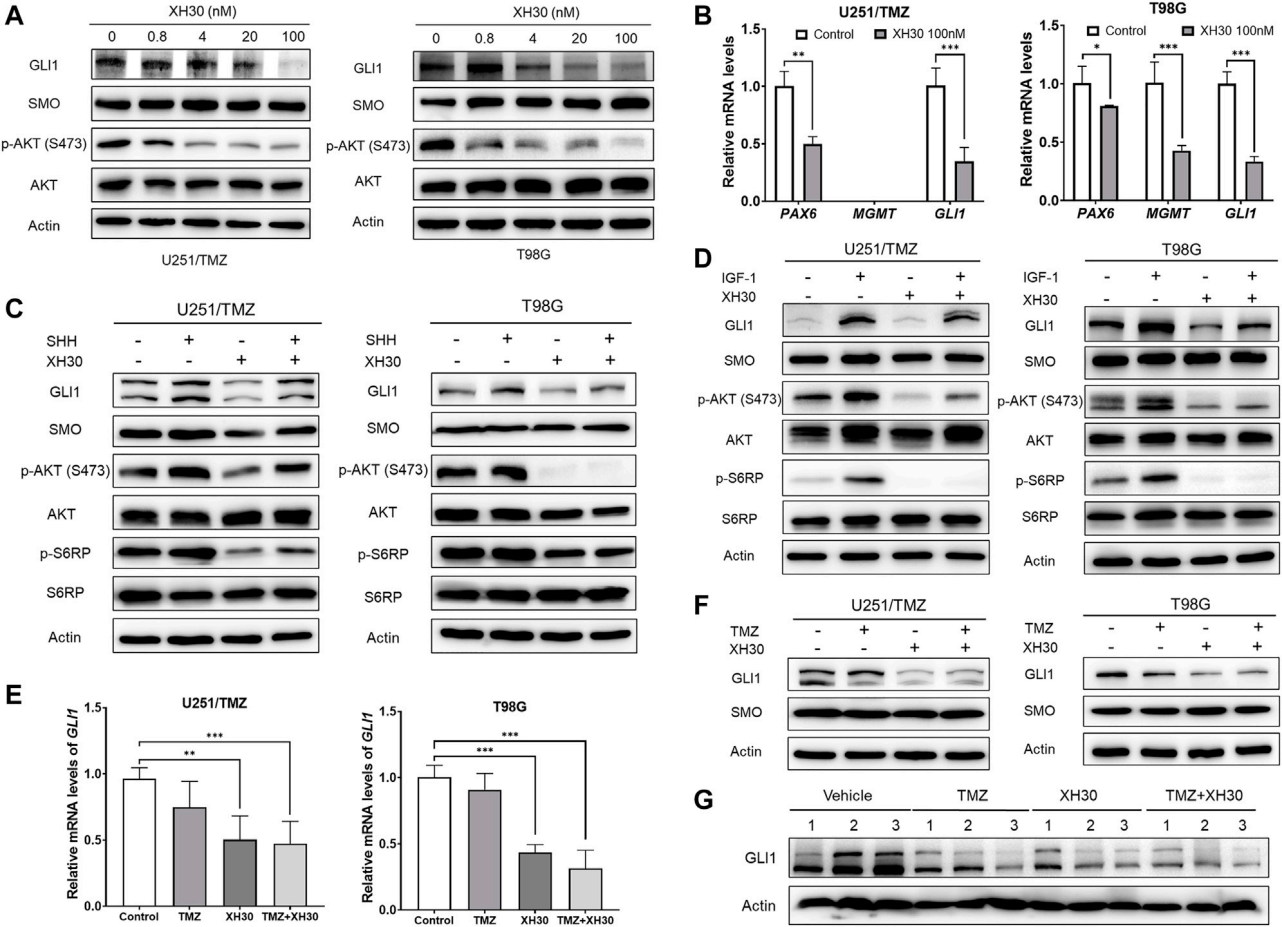
XH30 suppressed GLI1 *via* blockade of the noncanonical hedgehog signal pathway. **(A)** XH30 dose-dependently reduced GLI1 protein levels in TMZ-resistant U251/TMZ and T98G cells. Cells were incubated with XH30 at indicated concentrations (0.8, 4, 20, and 100 nmol/L) for 12 h **(B)** XH30 downregulated the GLI1 target gene expression, as indicated by mRNA levels, in both U251/TMZ and T98G cells. Data are presented as means ± SD, *n* = 3. *t*-test, ^*^ *p* < 0.05, ^**^ *p* < 0.01, and ^***^ *p* < 0.001, compared with control. **(C)** XH30 attenuated SHH-triggered GLI1 activation in U251/TMZ and T98G cells. The cells were incubated with XH30 at a concentration of 100 nM in the absence or presence of recombinant SHH (500 ng/ml) for 24 h. **(D)** XH30 reversed GLI1 activation by IGF-1 in U251/TMZ and T98G cells. Cells were incubated with XH30 at a concentration of 100 nM in the absence or presence of recombinant IGF-1 (50 ng/ml) for 12 h. **(E)** XH30 and TMZ in combination maintained lower mRNA levels of *GLI1* in GBM cells. The cells were incubated with XH30 (100 nM) or TMZ (250 μM) for 12 h. *t*-test, ^**^ *p* < 0.01 and ^***^ *p* < 0.001, compared with control. **(F)** XH30 and TMZ in combination maintained lower protein levels of GLI1 in GBM cells. The cells were incubated with XH30 (100 nM) or TMZ (250 μM) for 12 h. **(G)** Protein levels of GLI1 in tumor tissue from the U251/TMZ xenograft mice model.

The hedgehog signaling pathway has been demonstrated to have a role in TMZ resistance (Li J. et al., 2016; Lee, 2016), and crosstalk between noncanonical hedgehog and PI3K signaling pathways has been documented (Riobo et al., 2006). Previously, we showed that the hedgehog pathway is overactive in both U251/TMZ and T98G cells (Ji et al., 2018). Therefore, we hypothesized that inhibition of PI3K may also inhibit the noncanonical hedgehog pathway to increase the response to TMZ. As shown in Figure 6A, the protein levels of GLI1, a key factor in the hedgehog signaling pathway, was dose-dependently decreased in U251/TMZ cells exposed to XH30 in various concentrations, along with decreased levels of phosphorylated AKT. SMO protein levels did not decrease after treatment with XH30. Similar results were also observed in T98G cells. GLI1 target genes, such as *PAX6* and *GLI1* itself, were downregulated in the presence of XH30 at 100 nM (Figure 6B), likely because XH30 suppresses noncanonical hedgehog signaling pathway *via* the blockade of PI3K. In T98G cells, another GLI1 target gene, *MGMT*, was also downregulated by XH30. Next, we investigated whether XH30 was able to block the hedgehog pathway in the presence of the hedgehog ligand SHH (sonic hedgehog). As shown in Figure 6C, SHH activated the hedgehog pathway in U251/TMZ and T98G, reflecting upregulated GLI1 expression, whereas XH30 reduced GLI1 expression. In the presence of SHH, XH30 partially reversed SHH-mediated GLI1 activation. The phosphorylation of downstream proteins in the PI3K pathway, including AKT and S6RP, was consistently decreased in cells exposed to XH30. Moreover, insulin-like growth factor 1 (IGF-1), which activates PI3K, also upregulated GLI1 expression, which can be attributed to the crosstalk between the PI3K signaling pathway and GLI1 (Figure 6D). In the presence of XH30, IGF-mediated elevation of GLI1 was partially attenuated. There was no obvious change in SMO protein levels. We also observed that XH30 and TMZ in combination maintained lower level of GLI1 expression in both mRNA and protein level in TMZ-resistant GBM cells (Figures 6E,F). Immunoblotting results of tumor tissues also showed that the protein levels of GLI1 decreased in XH30 and TMZ combination group compared to vehicle group (Figure 6G). Together, our results demonstrate that XH30 suppressed GLI1 *via* blockade of the noncanonical hedgehog signaling pathway.

## Discussion

Glioblastoma is the most aggressive cancer of the brain in adults, and its prevalence is increasing, especially in China (Yang et al., 2013). Standard treatment includes surgery combined with radiotherapy and chemotherapy (Weller et al., 2014). So far, TMZ is the only chemotherapeutic option with confirmed efficacy and an acceptable safety profile in this cancer type (Rajaratnam et al., 2020). In addition, vascular endothelial growth factor inhibitors and tumor-treating fields have also been approved for the treatment of glioblastoma (Lassman et al., 2020; Seystahl et al., 2020; Zhang et al., 2020). However, patient outcomes from these treatments remain contentious. Clinical trials of immunotherapy with anti-programmed cell death 1 antibodies also did not meet clinical survival endpoints in patients with GBM (Khasraw et al., 2020; Maghrouni et al., 2021). Overactivation of the PI3K pathway occurs frequently in GBM, including in those with TMZ resistance (Sami and Karsy, 2013). In our previous study, XH30 exerted robust antitumor activity against TMZ-sensitive glioblastoma (Lin et al., 2018). Therefore, in this study, we tested this compound in GBM with either natural or acquired TMZ resistance. As expected, XH30 was shown to have acceptable antitumor activity against TMZ-resistant GBM both *in vitro* and *in vivo*, *via* inhibition of PI3K and downstream proteins and induction of cell cycle arrest. Similarly to other pan-PI3K inhibitors, we observed reductions in total white blood cells, neutrophils, and lymphocytes after XH30 treatment in our *in vivo* mouse model (Supplementary Figure S2B).

After standard treatment, GMB recurs in most people with this tumor type; TMZ resistance is a primary factor contributing to this process (Bocangel et al., 2002). For recurring tumors, repeated treatment with low doses of TMZ or treatment with lomustine (CCNU) has been recommended (Birzu et al., 2020; Di Nunno et al., 2020; Weller and Le Rhun, 2020). However, the clinical benefits of this approach are limited. The mechanism of TMZ resistance is extremely complex and includes overexpression of MGMT, aberrant activation of the hedgehog signaling pathway, overexpression of P-glycoprotein, and even metabolic reprogramming (Lee, 2016). Many approaches for overcoming TMZ resistance have been assessed (Happold and Weller, 2015), but none have been particularly successful. The hedgehog signal pathway represents an attractive target for glioblastoma treatment; inhibition of this pathway could overcome TMZ resistance (Shahi et al., 2008; Braun et al., 2012). In this context, a particularly interesting finding in our study is that XH30 dose-dependently decreased GLI-1 protein levels and downregulated its target genes including *PAX6* and *GLI1* itself in both U251/TMZ and T98G. In T98G cells, the mRNA level of *MGMT*, which may be regulated by GLI1, was reduced after XH30 treatment as well. This observation triggered us to explore the possible mechanism of XH30 as part of the hedgehog signaling pathway. There have been reports that PI3K signaling pathway is an important non-canonical activator of GLI1 and that targeting the PI3K/AKT pathway *via* GLI inhibition enhances drug sensitivity (Liang et al., 2017). Conversely, GLI1 reduces drug sensitivity *via* direct activation of the PI3K pathway in acute myeloid leukemia (Zhou et al., 2021). In our study, in the presence of SHH, XH30 suppressed the hedgehog pathway and partially reversed GLI1 activation. In addition, when we added IGF-1 to activate the PI3K signaling pathway, we found that GLI1 protein levels increased after IGF-1 stimulation. In the presence of XH30, this GLI1 level increase was partially attenuated. These results provided us with an additional clue that XH30 may play other roles in GBM treatment, which warrants further research.

Previous research has reported that inhibition of hedgehog signal pathway can enhance TMZ cytotoxicity and overcome TMZ resistance (Li J. et al., 2016; Ji et al., 2018). Therefore, we predicted that XH30 may also increase the response to TMZ *via* blockade of the non-canonical hedgehog pathway, allowing direct antitumor activity against TMZ-resistant GBM. In our experiments, XH30 enhanced the cytotoxicity of TMZ in both TMZ-resistant cell types *in vitro*. Treatment with combined XH30 and TMZ increased the level of λH2AX, a marker of DNA damage. Our *in vivo* studies in orthotopic mice also confirmed this synergistic effect. The combination of XH30 with TMZ yielded an improved antitumor activity compared with XH30 or TMZ treatment alone. This suggests that PI3K inhibitors could be tested as adjuvant treatment along with TMZ in patients with recurrent GMB.

In conclusion, the PI3K inhibitor XH30 exhibited robust antitumor activity in TMZ-resistant GBM; this compound is therefore a novel potential therapeutic option for TMZ-resistant GBM.

## Data Availability

The original contributions presented in the study are included in the article/Supplementary Material; further inquiries can be directed to the corresponding authors.
