# Supplementary material for: The PI3K Inhibitor XH30 Enhances Response to Temozolomide in Drug-Resistant Glioblastoma via the Noncanonical Hedgehog Signaling Pathway
Source: Front Pharmacol. 2021 Nov 26;12:749242. doi: 10.3389/fphar.2021.749242 (PMC8662317; doi:10.3389/fphar.2021.749242)
Supplement: Supplementary file 1 [file DataSheet1.docx]

**Supplementary Material**


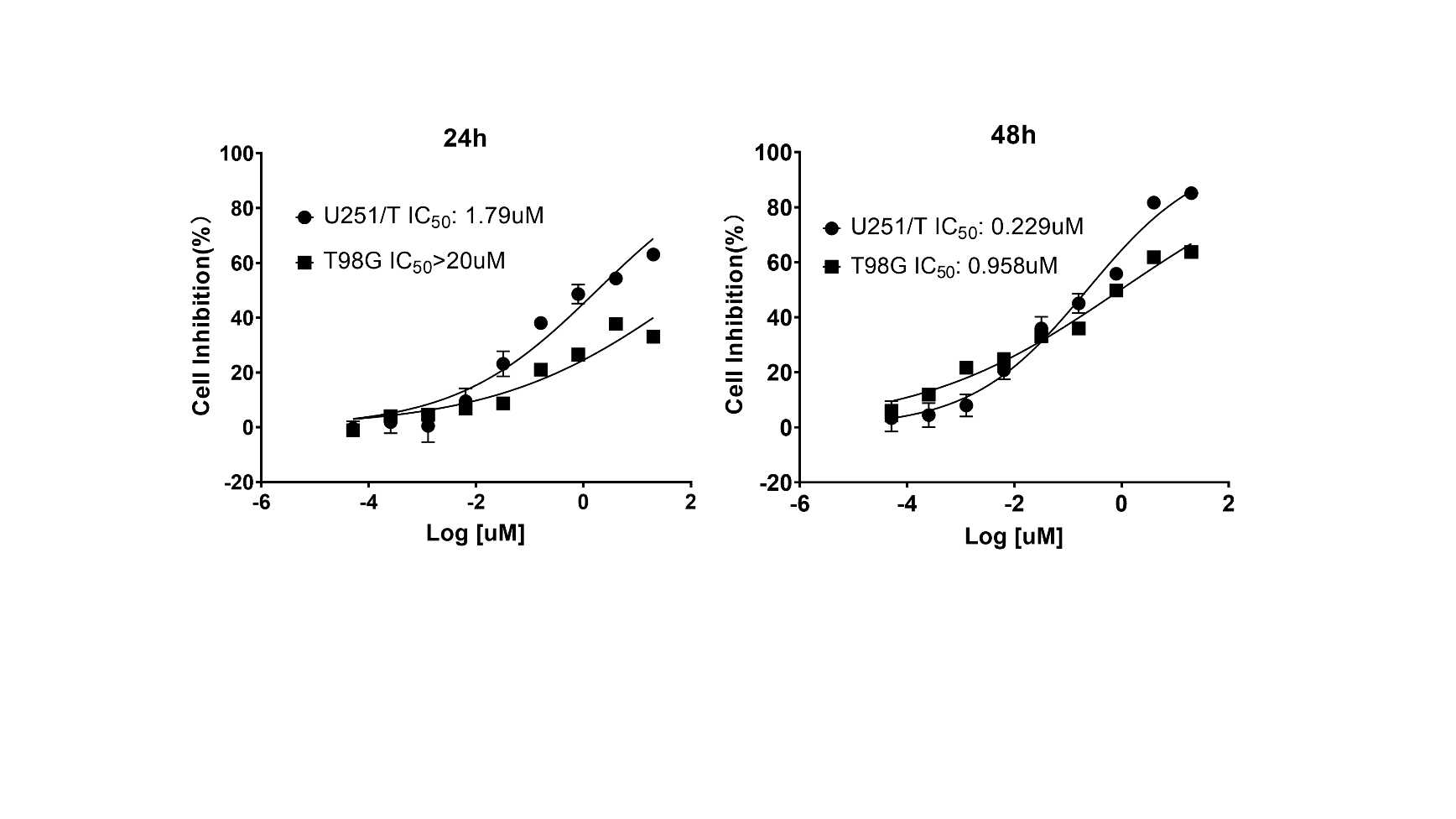


**Figure S1. The cytotoxicity of XH30 in temozolomide (TMZ)-resistant U251/TMZ and T98G cell lines at 24 h and 48 h.** Cells were seeded in a 96-well plate (8000 cells/well for 24 h and 4000 cells/well for 48 h). After incubation overnight, the cells were treated with different concentrations of XH30 for 24 h or 48 h. Then, the absorbance values were measured using the CCK-8 method.


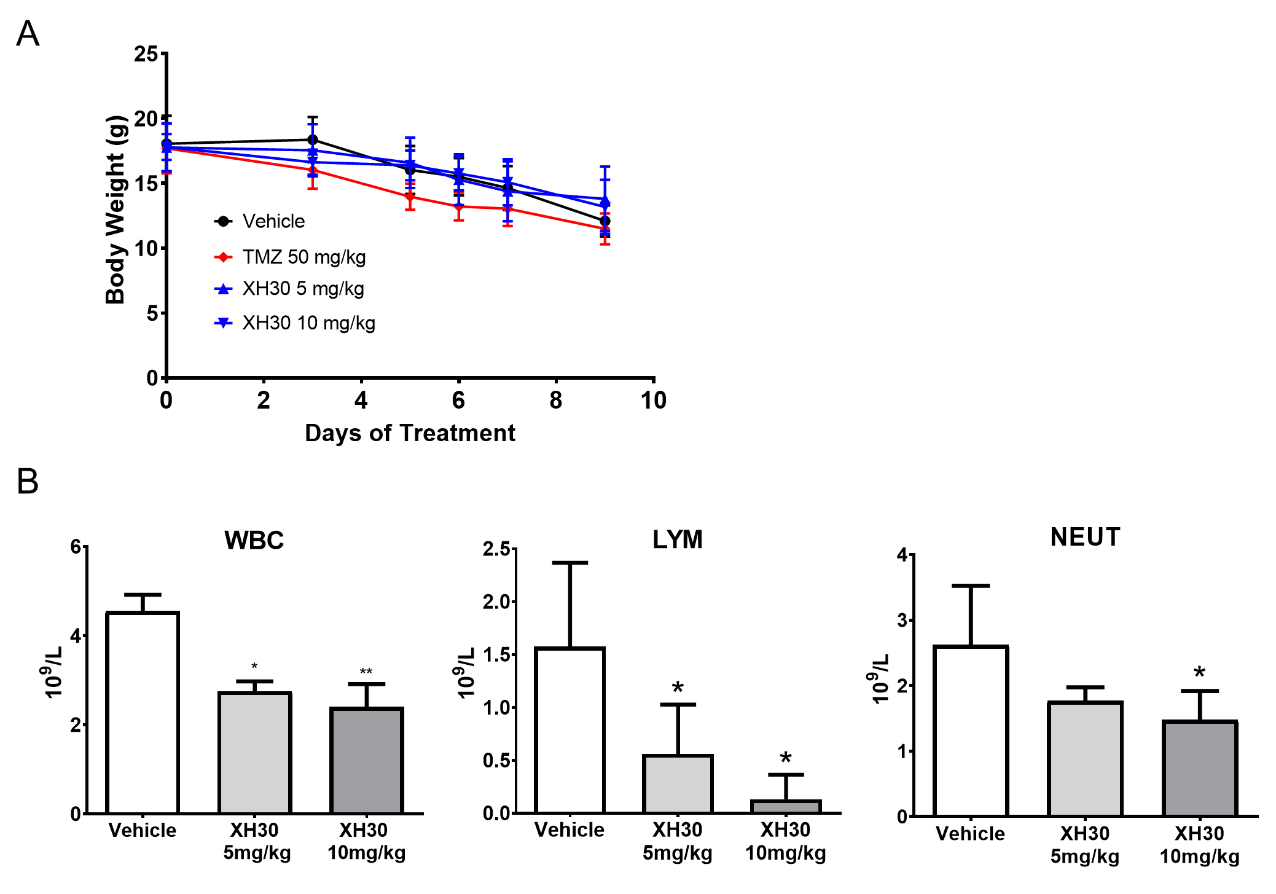


**Figure S2. XH30 repressed U251/TMZ growth in a mouse orthotopic xenograft model.** (A) The body weight of mice in the U251/TMZ orthotopic model. Data are presented as mean ± standard deviation (SD), n = 5. (B) Cell counts for white blood cells (WBC), neutrophils (NEUT) and lymphocytes (LYM) 2 h after last dosing. Analysis of variance (ANOVA) **p* < 0.05 compared with vehicle control group. Data are presented as mean ± SD, n = 5.


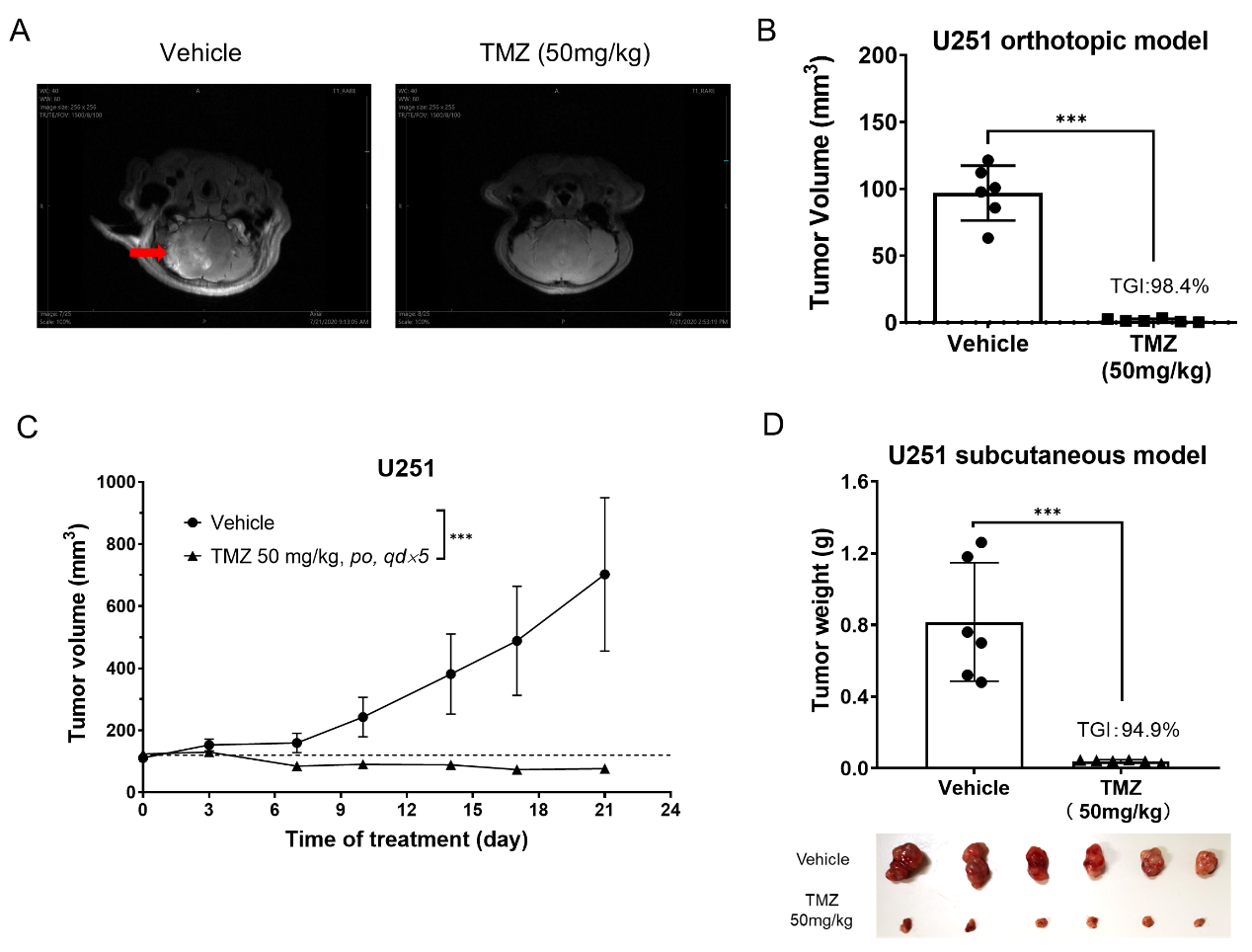


**Figure S3. Antitumor activity of temozolomide (TMZ) in a U251 xenograft mouse model.** (A) Representative magnetic resonance images (MRIs) from the U251 orthotopic model. The red arrow indicates a tumor. (B) Tumor volume in the U251 orthotopic model. T-test ^***^*p* < 0.001 compared with the vehicle control group. (C) Antitumor activity of TMZ in the U251 subcutaneous xenograft model. T-test, ^***^*p* < 0.001. Data are presented as mean ± standard deviation (SD), n = 6. TMZ was orally administered at the dose of 50 mg/kg for 5 days and effects were observed up to day 21. (D) Tumor weight in the U251 subcutaneous xenograft model. T-test, ^***^*p* < 0.001.


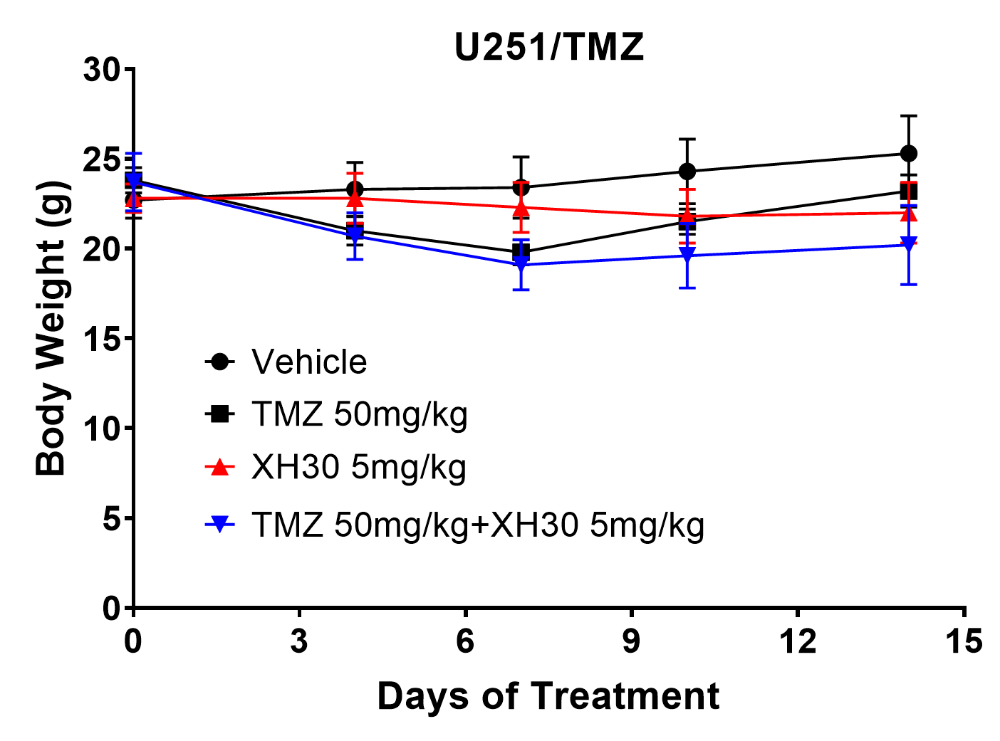


**Fig S4. The body weight of mice in a U251/TMZ subcutaneous xenograft model.** Data are presented as mean ± standard deviation (SD), n = 6.

**Table S1. The Antitumor activity of XH30 in a U251/TMZ orthotopic mouse model.**

| **Group** | **Dose**  **(mg/kg×days)** | **Mice number**  **(Day 0/Day 9)** | **Body Weight (g)** | | **Tumor Volume (mm^3^)** | |
| --- | --- | --- | --- | --- | --- | --- |
|  |  |  | **Day 0** | **Day 9** | **Mean ± SD** | **TGI%** |
| Vehicle | - | 5/5 | 18.1 ± 2.2 | 12.1 ± 1.2 | 206.3 ± 93.3 | - |
| TMZ | 50 × 5, *po, qd* | 5/5 | 17.7 ± 1.9 | 11.5 ± 1.2 | 193.5 ± 120.0 | 6.2 |
| XH30 | 5 × 9, *po, qd* | 5/5 | 17.8 ± 1.8 | 13.8 ± 2.5 | 116.1 ± 34.3 | 43.7 |
| XH30 | 10 × 9, *po, qd* | 5/5 | 17.8 ± 1.0 | 13.2 ± 2.1 | 25.0 ± 15.6^**^ | 87.9 |

Analysis of variance (ANOVA) ^**^*p*<0.01 compared with the vehicle control. *po,* delivered orally; *qd,* once a day; SD, standard deviation; TGI, tumor growth inhibition.

**Table S2. Combination index of XH30 and temozolomide (TMZ) in TMZ-resistant glioblastoma multiforme (GBM) cells.**

| **Cell lines** | **Combination index (CI)** |
| --- | --- |
| U251/TMZ | 0.625 |
| T98G | 0.234 |

Additive effect (CI = 1), synergism (CI < 1) and antagonism (CI > 1)

**Table S3. Antitumor activity of XH30 combined with temozolomide (TMZ) in a U251/TMZ subcutaneous xenograft model.**

| **Group** | **Dose** | **Mice number** | **Body weight (g)** | |  | **Tumor volume (mm^3^)** | | | | |  | | | **Tumor weight (g)** | |
| --- | --- | --- | --- | --- | --- | --- | --- | --- | --- | --- | --- | --- | --- | --- | --- |
|  | **(mg/kg×days)** | **(Day 0/Day 14)** | **Day 0** | **Day 14** |  | | **Day 0** | **Day 14** | | **T/C %** | |  | **Mean ± SD** | | **TGI (%)** |
| Vehicle | NA | 6/6 | 22.7 ± 1.0 | 25.3 ± 2.1 |  | | 102.5 ± 19.9 | 1729.9 ± 371.8 | NA | | |  | 2.53 ± 0.44 | | NA |
| TMZ | 50 × 5, *po, qd* | 6/6 | 23.8 ± 0.7 | 23.2 ± 0.9 |  | | 99.5 ± 2.8 | 782.5 ± 78.5 | 45.3 | | |  | 1.04 ± 0.14^***^ | | 58.9 |
| XH30 | 5 × 14, *po, qd* | 6/6 | 22.8 ± 0.8 | 22.0 ± 1.7 |  | | 95.2 ± 7.0 | 681.0 ± 213.6 | 39.4 | | |  | 0.76 ± 0.24^***,^ | | 69.9 |
| XH30+TMZ | 5 × 14, *po, qd;*  50 × 5, *po, qd* | 6/6 | 23.7 ± 1.6 | 20.2 ± 2.2 |  | | 100.3 ± 5.5 | 217.0 ± 60.0 | 12.6 | | |  | 0.25 ± 0.12^***^ | | 90.1 |

ANOVA analysis, ^***^*p*<0.001, compared to Vehicle. *po,* delivered orally; *qd,* once a day; SD, standard deviation; T/C%, the tumor volume of test group/the tumor volume of vehicle group; TGI, tumor growth inhibition.
